# Effects of interferon beta-1b on cognitive performance in patients with a first event suggestive of multiple sclerosis

**DOI:** 10.1177/1352458512442438

**Published:** 2012-10

**Authors:** Iris-Katharina Penner, Brigitte Stemper, Pasquale Calabrese, Mark S Freedman, Chris H Polman, Gilles Edan, Hans-Peter Hartung, David H Miller, Xavier Montalbán, Frederik Barkhof, Dirk Pleimes, Vivian Lanius, Christoph Pohl, Ludwig Kappos, Rupert Sandbrink

**Affiliations:** 1 University of Basel, Switzerland.; 2 University Hospital Basel, Switzerland.; 3 Bayer HealthCare Pharmaceuticals, Berlin, Germany.; 4 Ottawa Hospital, Canada.; 5 Vrije Universiteit Medical Center, The Netherlands.; 6 Clinique Neurologique, Rennes, France.; 7 Department of Neurology, Heinrich Heine Universität, Germany.; 8 National Hospital for Neurology and Neurosurgery, UK.; 9 Hospital Vall d’Hebron, Spain.; 10 Department of Neurology, University of Erlangen-Nürnberg, Germany.; 11 Department of Neurology, University of Bonn, Germany.

**Keywords:** demyelinating autoimmune diseases, CNS, multiple sclerosis, cognition, clinically isolated syndrome, interferon beta-1b, clinical trial

## Abstract

**Background:** Cognitive dysfunction occurs at the earliest stages of multiple sclerosis (MS), including the stage of clinically isolated syndrome (CIS).

**Methods:** We evaluated the impact of interferon beta-1b (IFNβ-1b) 250 µg on cognitive performance during the CIS stage in the BENEFITstudy. Cognition was assessed by Paced Auditory Serial Addition Test-3” (PASAT-3”) scores.

**Results:** Improvement in PASAT-3” score from baseline to year two was greater for IFNβ-1b treatment than placebo in patients not reaching clinically definite MS (CDMS) by year two. The treatment effect was maintained at year five and was statistically significant.

**Conclusions:** To conclude, early IFNβ-1b treatment had a sustained positive effect on PASAT-3” score over the 5-year BENEFIT study.

## Introduction

Cognitive changes occur in patients with all forms of multiple sclerosis (MS),^1,2^ including those presenting with a first event suggestive of MS known as clinically isolated syndrome (CIS).^3,4^ Even at this very early disease stage, cognitive changes have been reported, with a prevalence ranging from 27 to 57% depending on the criteria used to indicate cognitive decline.^2–5^ Cognitive impairment in CIS is already known to affect core domains of cognition, including working memory,^2,4^ complex attention,^2,3^ concentration,^2,3^ executive function^4^ and speed of information processing.^2,4^

Early initiation of disease-modifying treatment (DMT) at the stage of CIS has been demonstrated to delay conversion to clinically definite MS (CDMS), and to positively affect clinical and MRI disease aspects.^6–9^ However, despite the high relevance of cognitive impairment during the course of MS, key trials assessing the effects of DMTs in patients with CIS have not examined cognitive performance as a primary outcome.^6,7,10,11^

Some variable results on the effects of DMTs and cognitive performance in patients with established relapsing–remitting MS have been reported: in one study similar to ours, patients treated with intramuscular interferon beta-1a (Avonex®) performed significantly better after two years on tests of information processing and memory than patients randomized to receive placebo.^12^ In another randomized, placebo-controlled, multicentre, phase III trial, no difference in therapeutic effect on cognitive functioning was detected when glatiramer acetate (Copaxone®) treatment was compared with placebo.^13^ This finding of no difference was confirmed after 10 years in a long-term follow-up study among patients receiving early versus delayed treatment with glatiramer acetate.^14^

Here we report results of a further analysis targeted on cognitive performance during the placebo-controlled (up to year two) and follow-up phases (up to year five) of the Betaferon in Newly Emerging MS for Initial Treatment (BENEFIT study), as assessed by means of the Paced Auditory Serial Addition Test-3” (PASAT-3”), a component of the Multiple Sclerosis Functional Composite (MSFC) battery of tests.^15^

## Methods

### Patients

Four hundred and sixty-eight CIS patients were enrolled in the BENEFIT study and were randomized (5:3) to receive either interferon beta-1b (IFNβ-1b) 250 µg subcutaneously every other day or placebo for two years or until a diagnosis of CDMS. Following conversion to CDMS, or at the end of the two years, patients who entered the follow-up phase were offered open-label IFNβ-1b treatment for up to five years after randomization. Individuals initially assigned to IFNβ-1b treatment comprised the early treatment group, those individuals initially randomized to receive placebo prior to IFNβ-1b treatment represented the delayed treatment group. Complete study design and procedures, as well as primary results, have been published elsewhere.^8,9^

### Paced Auditory Serial Addition Test-3”

The current analysis examined PASAT-3” data collected during the double-blind and open-label/follow-up phases of the BENEFIT study. To reduce practice effects during the study, patients were tested at screening, tested once during the period between screening and baseline visit (the visit when therapy was initiated), and tested once at baseline visit. Following baseline testing, PASAT-3” was performed every six months throughout the 5-year study duration.^9^

### Statistics

Statistical analyses were performed post hoc and were exploratory in nature, as cognition was not a primary study outcome. In addition to descriptive statistics, non-parametric analysis of covariance (ANCOVA) was used for group comparisons with baseline PASAT-3” score as the covariate. PASAT-3’’ score changes from baseline were analysed at year two (placebo-controlled dataset) and at year five (integrated dataset of the placebo-controlled and open-label/follow-up phases). In addition, the dataset was analysed with the last observed PASAT-3” score post-baseline carried forward for all patients in the total population, using the last observation carried forward (LOCF) approach.

Patients were also stratified on the basis of baseline PASAT-3” scores into low and high cognitive performance groups. As no normative data in either healthy controls or patients with CIS are available for repeated PASAT-3” test scores an arbitrary threshold was chosen that still allowed for reasonable group sizes. This threshold was a PASAT-3” score of 52, since 33% of the total study population had PASAT-3” scores below this value.

### Standard protocol approvals, registrations and patient consents

Written informed consent was obtained from all individuals participating in the placebo-controlled and follow-up phases of the BENEFIT study (ClinicalTrials.gov Identifier NCT00185211).

## Results

### Patients

The demographic and disease characteristics recorded at the start of the BENEFIT trial for the 468 CIS participants are presented in Table 1. The majority of study participants had high baseline PASAT-3” scores (median = 55.0, mean ± SD = 52.6 ± 8.0), showing an increase from a median score of 49.0 (mean ± SD = 46.4 ± 10.5) at screening. Mean baseline cognitive performance in patients receiving IFNβ-1b (*n* = 290) and placebo (*n* = 174) treatments were similar, with mean PASAT-3” scores of 52.4 and 52.8 respectively. The distribution of PASAT-3” scores was highly negatively skewed, thus the mean was lower than the median, giving a right-steep distribution towards the upper limit of the maximum PASAT-3" score of 60. More than 75% of subjects had baseline scores ≥ 50 and 31% had baseline scores ≥ 58.

**Table 1.** Demographic and disease characteristics of early- and delayed treatment patients participating in the BENEFITstudy.

	Early treatment (*n* = 292)	Delayed treatment (*n* = 176)
Women, *n* (%)	207 (70.9)	124 (70.5)
Age, years, mean (median)	30.8 (30.0)	30.7 (30.0)
EDSS at screening, mean (median)	1.9 (2.0)	1.8 (2.0)
EDSS at baseline, mean (median)	1.6 (1.5)	1.5 (1.5)
PASAT-3” score at screening, mean (median)	46.5 (49.0)	46.2 (48.5)
PASAT-3” score at baseline, mean (median)	52.4 (55.0)	52.8 (55.0)
Patients with >9 T2 lesions at baseline, *n* (%)	207 (70.9)	123 (69.9)
Patients with ≥1 gadolinium-enhancing lesion at baseline, *n* (%)	127 (43.5)	70 (39.9)

EDSS, Expanded Disability Status Scale; PASAT-3”, Paced Auditory Serial Addition Test-3”

### Placebo-controlled phase

At the end of year two, 281 of the 285 participants who had completed the placebo-controlled study phase without progressing to CDMS underwent PASAT-3” assessment (placebo, *n* = 88; IFNβ-1b, *n* = 193). Overall median and mean ± SD PASAT-3” scores in this subgroup of patients were 57.0 and 54.6 ± 7.4. The change in PASAT-3” score from baseline to the end of year two was available for 279 patients: IFNβ-1b treatment patients had a larger mean increase in PASAT-3” score, indicating better cognitive performance, than placebo patients (Table 2).

**Table 2.** Change in PASAT-3” scores from baseline through the placebo-controlled phase and the follow-up phase.

Placebo-controlled phase	Year 2^a^	Year 2 (LOCF)^b^
IFNβ-1b treatment^c^	2.3± 6.3, 1.0 (0 to 5.00) (*n* = 191)	2.0 ± 6.3, 1.0 (–1.0 to 4.0) (*n* = 273)
Placebo treatment^c^	0.8 ± 5.5, 0.5 (–1.0 to 3.0)) (*n* = 88)	0.6 ± 5.4, 0.5 (–1.0 to 3.0) (*n* = 166)
*p* value^d^	0.018	0.021
Follow-up phase	Year 5	Year 5 (LOCF)
Early treatment^c^	3.4 ± 6.2, 2.0 (0 to 6.0)) (*n* = 227)	3.0 ± 6.0, 2.0 (0 to 6.0) (*n* = 285)
Delayed treatment^c^	1.5 ± 6.8, 1.0 (–1.0 to 4.0)) (*n* = 120)	1.0 ± 6.5, 1.0 (–1.0 to 4.0) (*n* = 174)
*p* value^d^	0.005	<0.001

a Includes only those patients who did not progress to CDMS until year 2.

b Includes patients who progressed to CDMS before year two or prematurely discontinued study.

c Data are mean ± SD, median (interquartile range).

d Non-parametric analysis of covariance for change in PASAT-3’’ score from baseline.

IFNβ-1b: interferon beta-1b; LOCF: Last observation carried forward; PASAT-3”: Paced Auditory Serial Addition Test-3”

Using the LOCF approach, the last scheduled, non-missing, post-baseline measurement obtained during the 2-year, placebo-controlled study period was analysed and included those patients who progressed to CDMS before year 2. This sensitivity analysis yielded a similar result: based on data from 439 patients, a difference in the change in PASAT-3” score from baseline to year 2 in favour of IFNβ-1b was found (Table 2).

### Follow-up phase

At year five, 349 patients had available PASAT-3” scores (delayed treatment, *n* = 120; early treatment, *n* = 229). From the end of the placebo-controlled phase until the end of the follow-up period at year five, PASAT-3” scores further increased slightly (median = 58.0; mean ± SD = 55.6 ± 6.5).

Cognitive improvements from baseline to year five were significantly more pronounced amongst the early treatment patients (*n* = 227), who had a mean 3.4 point (median = 2.0) increase in PASAT-3” score from baseline to year five. Patients in the delayed treatment group (*n* = 120) had a mean 1.5-point (median = 1.0) increase in score; a change from baseline that was significantly less than that measured for the early treatment group (*p* = 0.005) (Table 2). This outcome was confirmed with the sensitivity analysis based on the LOCF approach, where the mean increases in PASAT-3” score from baseline to year five were 3.0 versus 1.0 in early- and delayed treatment groups respectively (*p* < 0.001) (Table 2).

There was no difference in PASAT-3’’ scores after five years in patients who progressed to CDMS (median = 58.0; mean ± SD = 55.3 ± 7.5) as compared to patients who did not progress (median = 58.0; mean ± SD = 56 ± 5.5; *p* = 0.57).

**Figure 1.**
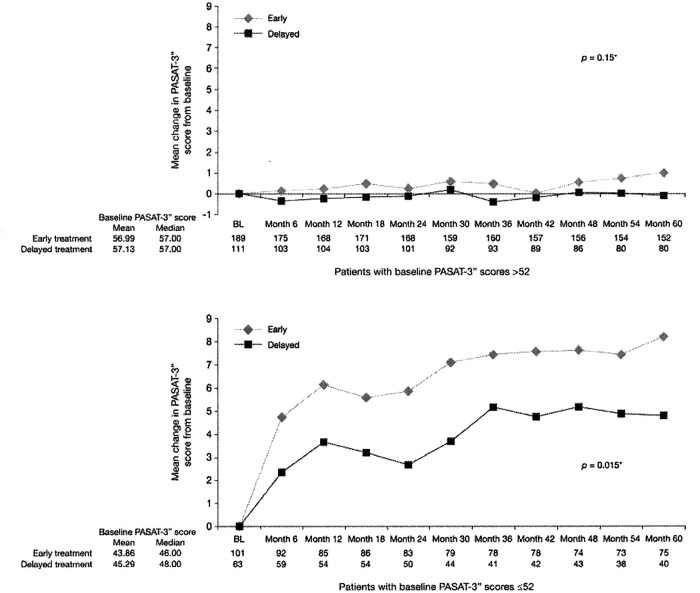
Relationship between treatment, PASAT-3” baseline performance and PASAT 3” improvement over 5 years. PASAT-3”:Paced Auditory Serial Addition Test-3”

Stratification of patients’ scores by baseline PASAT-3” score performance revealed distinct treatment effects. Figure 1 illustrates that there was a pronounced treatment effect on PASAT-3” performance in those patients with lower baseline scores (i.e. those patients with a greater potential to increase their PASAT-3” performance). Although the size of this subgroup was small, the treatment effect was robust (year five, *p* = 0.015; year five LOCF, *p* = 0.002) (Figure 1). Of note, an increase in PASAT-3” performance was also observed in patients with high baseline scores. Although statistical significance was not achieved, this increase was numerically more pronounced in early treatment patients (year five, *p* = 0.15; year five LOCF, *p* = 0.08) (Figure 1).

## Discussion

Our results indicate that initiation of treatment with subcutaneous IFNβ-1b, starting at the time of the first event suggestive of MS, has a favourable effect not only on disease activity,^9^ but also on cognitive performance as measured by PASAT-3” scores. During the placebo-controlled phase of the BENEFIT study, increases in PASAT-3” scores were observed in both treatment arms, indicating that some improvement in cognitive performance occurred independently of treatment assignment, an effect known to occur with repeated PASAT-3" administration.^16^ However, IFNβ-1b treatment did enhance this improvement: patients randomized to active treatment experienced significantly greater increases in PASAT-3” score from baseline to year two than did patients assigned to the placebo group.

The BENEFIT study was designed not only to evaluate the effects of IFNβ-1b treatment relative to placebo in patients with CIS, but also to characterize the impact of initiating treatment earlier rather than later after the first manifestation of MS.^8^ Regarding cognitive performance, we found that the increase in PASAT-3” score from baseline to year five remained significantly larger in early than delayed treatment patients. Thus, the benefits of early treatment with IFNβ-1b on PASAT-3" performance achieved during the first two years were maintained during the follow-up phase of the trial.

To minimize practice effects during the trial, patients in the BENEFIT study performed PASAT-3” assessment twice before the acquisition of the baseline measurement. This strategy resulted in a mean 6-point score increase from screening (46.4) to baseline (52.6). As a consequence, the mean PASAT-3” baseline score for the CIS patients enrolled in the BENEFIT study was higher than that previously reported for a control group, where the mean PASAT-3” score was 47.2.^17^ Given that PASAT-3” performance further improved during the trial, a ceiling effect had to be taken into consideration. To assess the impact of treatment in the light of this ceiling effect, we stratified patients into subgroups based on ‘low’ and ‘high’ baseline PASAT-3” values (≤52 versus >52). The choice of this cut-off value was somewhat arbitrary as no adequate normative data were available for repeated PASAT-3" assessments. Although, given this limitation, a cautious interpretation is due, the results seem to indicate that the treatment effects were more pronounced in patients with lower baseline PASAT-3” scores than those with higher scores, suggesting that total treatment effects might have been partially masked by the exceptionally high baseline performance of many patients.

Cognitive impairment in MS may have widespread detrimental effects on patients’ lives. Patients with MS who are cognitively impaired are more likely to become unemployed, to experience a decline in living standards and to have limited social lives.^18,19^ Given these grave consequences, it is important to prevent cognitive decline.
